# Postextubation Respiratory Muscle Recovery After Sugammadex or Neostigmine: A Randomized Controlled Ultrasonographic Trial in Laparoscopic Cholecystectomy

**DOI:** 10.1155/anrp/3991360

**Published:** 2026-09-28

**Authors:** Mohamed A. F. Ghoneim, Asmaa AboBakr AbdAllah Mohamed, Laila Ali El-Said El-Kafrawy, Ashraf Mahmoud Hazem Mohamed, Ahmed Wageh Ezzat Desoky

**Affiliations:** ^1^ Anesthesia, Intensive Care and Pain Medicine Department, Faculty of Medicine, Ain Shams University, Cairo, Egypt, asu.edu.eg

**Keywords:** comparison of sugammadex and neostigmine in improving expiratory muscle function during laparoscopic cholecystectomy

## Abstract

**Background:**

Sugammadex minimizes the residual neuromuscular block risk. By enhancing the function of expiratory muscles, it improves the clearance of secretions and helps prevent lung collapse, leading to a reduction in early postoperative respiratory issues. The study aims to compare the efficacy of sugammadex in promoting the recovery of expiratory muscle power to that of neostigmine following extubation.

**Study Objectives:**

The main objective of this study was to evaluate the effectiveness of sugammadex and neostigmine as a reversal of neuromuscular block, assessed through ultrasonographic evaluation of expiratory muscle strength, specifically the thickening fraction of the internal oblique muscle (TFIO). The secondary objective was to assess diaphragmatic excursion (DE) and to explore the usefulness of ultrasonography in comparison to train‐of‐four (TOF) monitoring.

**Patients and Methods:**

The study involved a total of 60 patients who underwent laparoscopic cholecystectomy between August 2023 and September 2024. Patients were randomly assessed into two groups for a comparative study. Group A received sugammadex, while Group B received neostigmine. The study utilized ultrasonography to measure the TFIO, reflecting expiratory muscle power, and DE, representing inspiratory muscle strength. Measurements were taken at three points in time: preinduction (baseline), at the TOF ratio recovery (TOFR) to 0.9, and 30 minutes postarrival in the postanesthesia care unit (PACU). The primary outcome was the variation in TFIO from baseline to a TOFR of ≥ 0.9. Secondary outcomes included the postoperative variation in DE from baseline, the incidence of recovery to baseline TFIO and DE, and the time taken for TOFR recovery to 0.95 and 1.

**Results:**

A significant difference in the primary outcome was observed among 60 patients. The alteration in TFIO from baseline to TOFR ≥ 0.9 was markedly reduced with sugammadex relative to neostigmine (mean ± SD: 9.13 ± 6.25 vs. 19.32 ± 5.75; adjusted *p* < 0.001). Sugammadex resulted in a significantly smaller reduction in DE from initial values to TOFR ≥ 0.9 compared to neostigmine (right side: 1.91 ± 0.66 vs. 2.34 ± 0.52; adjusted *p* < 0.05). Thirty minutes post‐PACU arrival, a greater percentage of patients in the sugammadex group reached baseline TFIO in comparison to the neostigmine group (67% vs. 47%); even so, the difference was not of statistical significance (corrected *p* value = 0.118). The rate of TFIO and DE returning to initial values remained low for both groups, even after TOFR reached 1. The sugammadex group exhibited a significantly accelerated recovery of neuromuscular function. The median recovery time for the TOFR from 0.9 to 0.95 was 0.32 min, compared to 1.74 min with neostigmine (corrected *p* < 0.001). The median recovery time from 0.9 to 1.0 was 0.53 min, compared to 5.13 min (corrected *p* < 0.001). The neostigmine group exhibited a higher incidence of postoperative complications, including atelectasis (16.7% vs. 10%), hypoxia (16.7% vs. 6.7%), tachycardia (40% vs. 0%), PONV (16.7% vs. 10%), recurarization (3.3% vs. 0%), and other adverse drug effects (10% vs. 6.6%). No cases of reintubation, airway obstruction, pulmonary edema, or postoperative residual paralysis were observed in either group.

**Conclusion:**

Sugammadex results in better expiratory muscle power recovery compared to neostigmine at TOFR of ≥ 0.9. These findings highlight that respiratory muscle function may remain compromised, although a TOFR of 1.0 is achieved.

**Trial Registration:** ClinicalTrials.gov identifier: NCT06432621

## 1. Introduction

Diaphragm ultrasound is a diagnostic tool used to evaluate respiratory function. The diaphragm, which is the chief muscle involved in breathing, is responsible for 60% of the tidal volume per breath. This method can help detect any residual dysfunction in the diaphragm muscle [1].

The reported postoperative residual curarization (PORC) incidence varies widely in the literature, ranging from approximately 20% to over 60%, depending on factors such as neuromuscular monitoring, type of surgery, and reversal strategy. In one study, the incidence was reported as 57.8% at extubation and 45.2% in the PACU [2]. The risk of postoperative pulmonary complications (PPCs) increases in cases of residual neuromuscular blockade (NMB) [3]. Residual paralysis may lead to reduced strength of the respiratory muscles and an impaired cough reflex. This may result in difficulties in sustaining an unobstructed upper airway and affect swallowing functions. In addition, it increases the incidence of lung collapse, decreases oxygen saturation, contributes to aspiration pneumonia, and results in complications with airway patency [4].

Sugammadex exhibits a reduced incidence of residual NMB compared to neostigmine [5]. The relationship with PPCs remains unclear [6]. Sugammadex exhibits superior efficacy in improving patients’ strength compared to neostigmine; however, the effects of various reversal agents on respiratory muscle power remain a subject of discussion [7]. Cappellini et al. showed that the diaphragmatic power recovery, which reflects inspiratory muscle function during extubation, was improved in patients who received sugammadex for reversal [8]. Prior work has suggested that sugammadex may enhance inspiratory muscle recovery, but the impact of different reversal agents on expiratory muscle strength and cough effectiveness remains incompletely defined [9]. Abola et al. showed that both have a nearly identical influence on forced vital capacity [7].

A recent study found that the TFIO, indicative of expiratory muscle function, correlates with the airway pressure generated during coughing. A reduction in the thickness of the abdominal muscles, observed during coughing, correlates with a higher incidence of extubation failure [10].

Building on this concept, TFIO and DE may provide a more direct evaluation of respiratory muscle recovery than traditional train‐of‐four (TOF) monitoring [11].

Unlike previous work conducted in microlaryngeal surgery, this trial focuses on patients undergoing laparoscopic cholecystectomy, a setting in which pneumoperitoneum and increased intra‐abdominal pressure may exert additional and sustained effects on diaphragmatic and abdominal wall mechanics. By combining quantitative TOF monitoring with ultrasonographic assessment of TFIO and DE, this study seeks to determine whether achieving conventional TOFR thresholds (≥ 0.9 and 1.0) is sufficient to restore respiratory muscle function in this specific surgical context. In addition, we explore sugammadex effect against neostigmine on early postoperative hemodynamic and pulmonary adverse events, including tachycardia and pulmonary complications [9].

### 1.1. Objective of the Study

The primary aim of the study was to compare sugammadex versus neostigmine efficacy in NMB reversal, as assessed by the recovery of expiratory muscle strength using ultrasonographic for measuring the TFIO.

The secondary aim was to evaluate inspiratory muscle recovery using DE and to explore the role of ultrasonography in the detection of neuromuscular blockade residual compared with traditional TOF monitoring.

## 2. Patients and Methods

### 2.1. Study Population

The study cohort consisted of adult patients who underwent laparoscopic cholecystectomy. Patients were randomly apportioned into two groups using a computer‐generated random number sequence and concealed opaque‐sealed envelopes:

1. Group A: patients received sugammadex (30 patients).
2. Group B: patients received neostigmine (30 patients).

### 2.2. Inclusion Criteria

1. Age 21–60 years.
2. Sex: Both sexes.
3. Patients with the American Society of Anesthesiology (ASA) classifications I and II.

### 2.3. Exclusion Criteria

1. ASA classifications III–V.
2. Impairment of renal function or kidney disease.
3. Impairment of hepatic function (Child–Pugh class B or C).
4. Chronic obstructive lung disease.
5. Pre‐existing neuromuscular disease.
6. History of cardiac arrhythmias or current intake of antiarrhythmic medications.
7. Known allergy or hypersensitivity to rocuronium, neostigmine, or sugammadex.
8. Predicted difficult airway (based on preoperative assessment).
9. Pregnancy or lactation.
10. Patient refusal to participate.
11. Body mass index (BMI) > 40 kg/m^2^ (morbid obesity).
12. Patients diagnosed with obstructive sleep apnea.
13. Known diaphragmatic paralysis.

**S**ample size: The sample size was calculated using PASS 15 software. With a power of 80% and an alpha error of 0.05, at least 20 patients per group were needed to show a statistically significant difference in the primary outcome, namely, the thickening fraction of the internal oblique muscle (ΔTFIO) change in the sugammadex versus neostigmine groups. This calculation was based on previously reported data by Huang et al. [12], which demonstrated a mean ΔTFIO of 9% ± 6% with sugammadex and 16% ± 9% with neostigmine.

Although the least sample size was 40 patients (20 per group), a total of 60 patients (30 per group) were enrolled to compensate for potential dropouts and to increase the statistical power and reliability of the results.

Ethical considerations: The study was approved by the local ethics committee (Faculty of Medicine, Ain Shams University). Written informed consent was signed by all patients before enrollment. The trial was prospectively registered at ClinicalTrials.gov (Identifier: NCT06432621).

### 2.4. Randomization and Allocation Concealment

Patients were allocated in a ratio of 1:1 to either the sugammadex group or the neostigmine group using a computer‐generated randomization sequence. Allocation concealment was ensured using sequentially numbered, opaque, sealed envelopes prepared by an investigator not involved in patient recruitment or data collection. Each envelope was opened immediately before receiving the reversal agent.

### 2.5. Blinding

The study design was a single‐blind randomized controlled trial. The investigator performing ultrasonographic measurements of DE and internal oblique muscle thickening fraction was blinded to group distribution.

Due to the intervention nature, the attending anesthesiologists administering the reversal agents were not blinded. Additionally, patients were not blinded, as no placebo or identical preparation of study drugs was used.

### 2.6. Protocol Changes

There were no modifications to the study design, eligibility criteria, interventions, outcomes, or analysis plan after trial commencement.

## 3. Study Procedures

### 3.1. Preoperative Settings

Preoperative assessment was done for all patients, including history taking regarding allergies to rocuronium, neostigmine, or sugammadex, a comprehensive medical history, ASA classification, and surgical history. A comprehensive physical examination involves vital signs (blood pressure, heart rate, SpO_2_), chest auscultation, cardiac and abdominal assessments, and laboratory tests CBC, coagulation profile, kidney and liver function, ECG for patients over 40 years, and ECHO for 60‐year‐old patients.

### 3.2. Intraoperative Settings

#### 3.2.1. Anesthetic Management

##### 3.2.1.1. Induction and Maintenance

Pulse oximetry, ECG, noninvasive blood pressure, and capnography were monitored. All patients received identical anesthetic management, including premedication with 2 mg of midazolam. Anesthesia was administered using propofol at a dosage of 1–2 mg/kg and fentanyl at 1–2 mcg/kg. Following the calibration of the TOF (NMT GE measurement setup utilizing a mechanosensor based on kinematography), rocuronium at a dosage of 0.6 mg/kg was administered, and intubation of patients occurred after a fade in the TOF count. Rocuronium at a dosage of 0.15 mg/kg and sevoflurane at a minimum alveolar concentration of 2 were utilized for the maintenance of anesthesia. Adjuvants to anesthesia comprised a 1‐mg intravenous infusion of granisetron for antiemetic purposes, a 30‐mg intravenous infusion of ketorolac for analgesia, and a 1‐g intravenous infusion of paracetamol. Cefazolin 1 g was administered prior to the skin incision.

##### 3.2.1.2. Neuromuscular Monitoring

Quantitative neuromuscular monitoring was conducted using a GE CARESCAPE B650 monitor with an NMT module featuring a mechanosensor based on kinemyography. The depth of NMB monitoring was determined by the device at the left adductor pollicis muscle. After skin preparation, two electrodes were placed at the wrist over the ulnar nerve. An acceleration transducer was affixed to the thumb distal phalanx. The device was calibrated with repeated TOF stimulation over 3 minutes until a stable TOF ratio between 0.9 and 1.1 was achieved. The TOF ratio was recorded every 15 s during the surgery and for 30 min postoperatively in the PACU. Rocuronium maintenance doses (0.15 mg/kg) were given when the post‐tetanic count reached ≥ 3.

### 3.3. Recovery

Pharmacological reversal was administered upon recovery to a TOF count of ≥ 1 and clinical signs of awakening. The reversal agents were administered as follows: Group A was given sugammadex (2 mg/kg IV), and Group B was given neostigmine (50 μg/kg IV, maximum 5 mg) coadministered with atropine (25 μg/kg IV, maximum 2.5 mg).

## 4. Outcome Measures and Data Collection

The TOF monitoring system was used to document the TOF ratio. Ultrasonography (Sonosite M‐Turbo) was used to assess the TFIO, which is indicative of expiratory muscle strength, and DE, reflecting inspiratory muscle strength. Measurements were obtained at three time points: before induction (baseline), at TOF ratio ≥ 0.9 (postextubation), and 30 min after arrival in the PACU. The operator was blinded to whether patients had received sugammadex or neostigmine. Patients were positioned semi‐recumbent, with the head elevated to approximately 45°. As this technique is effort‐dependent, the maximum value from three consecutive measurements was recorded.

A high‐frequency linear (HFL) 50x probe (bandwidth 6–15 MHz; scan depth 6 cm) was used to assess expiratory muscle strength. The probe was applied perpendicularly to the right anterior axillary line at the halfway point between the inferior costal margin and the iliac crest. The abdominal muscles appeared as hypoechogenic layers encircled by a hyperechoic fascial sheath. The internal oblique muscle thickness was recorded at end‐inspiration and during coughing, representing peak muscle contraction (Figures 1, 2). TFIO was calculated as the percentage change in thickness during coughing by the following formula: (TFIO = [end‐coughing thickness – end‐inspiratory thickness]/[end‐inspiratory thickness] × 100%) [9].

**FIGURE 1:**
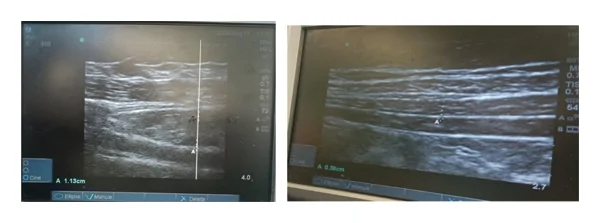
Internal oblique end of coughing; internal oblique dimension view end of inspiration with sugammedex TOFR 0.9. Abbreviation: TOFR, train‐of‐four ratio.

**FIGURE 2:**
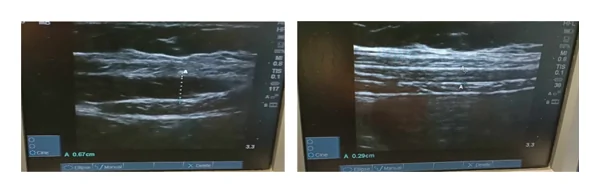
Internal oblique end of coughing; internal oblique dimension view end of inspiration with neostigmine at TOFR 0.9. Abbreviation: TOFR, train‐of‐four ratio.

The C60xi convex transducer (bandwidth: 2–5 MHz, depth: 30 cm) was used to assess DE during maximum sniff breathing. The transducer was positioned at the midclavicular line beneath the right costal arch, with the probe directed cranially and perpendicular to the cupula of the diaphragm. The diaphragm was selected as a line of echogenicity delineating the liver from the lung through B‐mode imaging. M‐mode was employed to assess diaphragm excursion (DE) on the vertical axis by tracing from the base to the apex during inspiration (Figures 3, 4). The speed was established at 25 mm/s to ensure the capture of at least three respiratory cycles within a single view.

**FIGURE 3:**
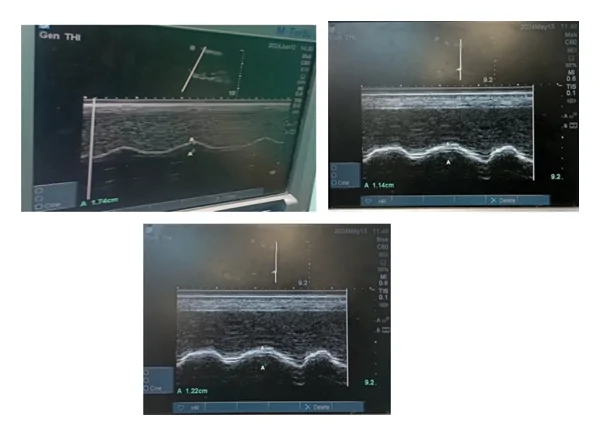
View of diaphragmatic excursion with sugammadex (baseline, at TOFR 0.9, and after 30 min in PACU). Abbreviations: TOFR, train‐of‐four ratio; PACU, postoperative care unit.

**FIGURE 4:**
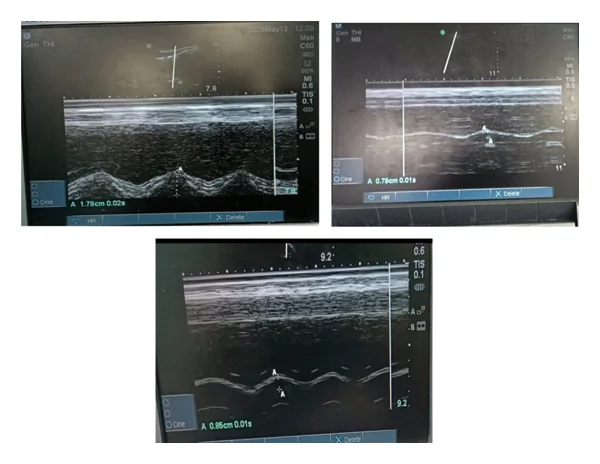
View of diaphragmatic excursion with neostigmine (baseline, at TOFR 0.9, and after 30 min in PACU). Abbreviations: TOFR, train‐of‐four ratio; PACU, postoperative care unit.

In Group A.

Patients were reversed with sugammadex at a dosage of 2 mg/kg.

In Group B.

Patients were reversed with neostigmine at a dosage of 50 μg/kg, maximum 5 mg.

### 4.1. DE Measurement

DE was assessed on the right hemidiaphragm only, as it provides more reliable and reproducible measurements due to the acoustic window offered by the liver.

Assessment of the left hemidiaphragm was not performed due to technical limitations related to gastric and bowel gas interference.

### 4.2. Measurements

#### 4.2.1. Primary Outcome

The impact of sugammadex and neostigmine on the change in TFIO from baseline (ΔTFIO) and the percentage change in DE from baseline (ΔDE%) to TOFR ≥ 0.9, a commonly accepted threshold for recovery from neuromuscular blockade, was studied.

The percentage change in DE from baseline (ΔDE%) was calculated as:

[(baseline DE − DE at the specified time point)/baseline DE] × 100.

#### 4.2.2. Secondary Outcomes

• The incidence of DE reverts to baseline levels at both TOFR ≥ 0.9 and after 30 min in the PACU.
• ΔTFIO to 30 min in the PACU.
• ΔDE to 30 min in the PACU.
• The incidence of TFIO returns to the baseline value at both TOFR ≥ 0.9 and after 30 min in the PACU.
• Time from TOFR of 0.9–0.95.
• Time from TOFR of 0.9–1.

The incidence of postoperative pulmonary problems (including atelectasis, hypoxia, and recurarization) and adverse effects of drugs (including tachycardia and postoperative nausea and vomiting) were also recorded as secondary outcomes.

The TFIO reflects expiratory muscle contractility during coughing, where higher values indicate stronger expiratory muscle function. The change in TFIO (ΔTFIO) was calculated as the percentage reduction from baseline values; therefore, larger ΔTFIO values indicate greater impairment of expiratory muscle strength, while smaller values reflect better preservation of muscle function.

It is calculated as:

TFIO (%) = (Thickness at end‐cough−Thickness at end‐inspiration)/Thickness at end‐inspiration × 100.

### 4.3. Interpretation

• Higher TFIO ⟶ stronger expiratory muscle contraction (better cough strength).
• Lower TFIO ⟶ weaker expiratory muscle function.

This is clinically important because expiratory muscles are essential for:

• Effective coughing
• Secretion clearance
• Atelectasis prevention and postoperative pulmonary complications.

## 5. Results

A total of 80 patients were assessed for eligibility. Of these, 20 patients were excluded, including 12 who did not meet the inclusion criteria and 8 who refused to participate. The remaining 60 patients were randomly allocated into two equal groups (*n* = 30 each). Group A received sugammadex, and all allocated patients received the intervention and were included in the analysis. Group B received neostigmine, and similarly, all patients received the allocated intervention and were analyzed. There were no losses to follow‐up or exclusions after randomization in either group, resulting in complete data analysis for all 60 participants (Figure 5).

**FIGURE 5:**
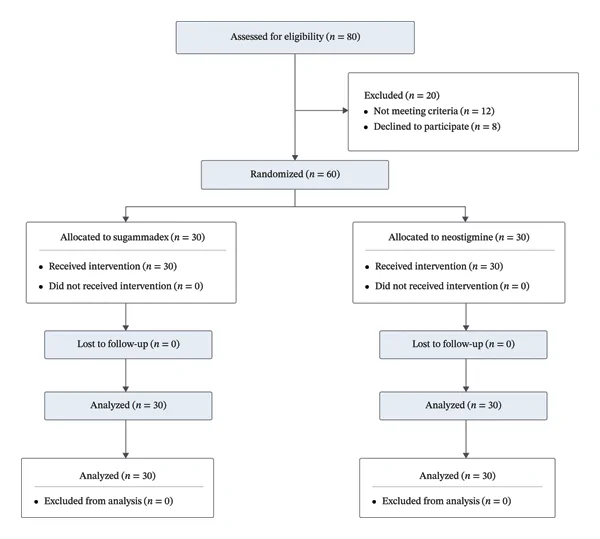
CONSORT flow diagram.

Table 1 demonstrates that the two groups are comparable across all recorded baseline characteristics, which supports the validity of subsequent outcome analyses.

(1) ΔTFIO %=TFIO baseline−TFIO at TOFR 0.930 or at  minTFIO baseline.

**TABLE 1:** Baseline characteristics among the studied groups.

	Sugammadex group (*n* = 30)	Neostigmine group (*n* = 30)	*p* value
	Mean ± SD	Mean ± SD	
Age	37.47 ± 5.83	38.6 ± 7.78	0.525

BMI	28.3 ± 3.61	27.86 ± 3.66	0.644

Operative duration	74.7 ± 5.69	75.73 ± 6.77	0.525

Preoperative Hb (gm/dL)	12.07 ± 0.81	11.07 ± 0.81	0.756

Preoperative creatinine (mg/dL)	0.94 ± 0.14	0.93 ± 0.18	0.849

Preoperative bilirubin (mg/dL)	1.02 ± 0.08	1.00 ± 0.07	0.387

Duration of pneumoperitoneum	47.7 ± 5.3	48.07 ± 6.8	0.817

Total rocuronium dose (mg/kg)	0.82 ± 0.11	0.83 ± 0.11	0.629

Time from last dose to reversal (min)	34.7 ± 5.69	35.73 ± 6.77	0.525

Total dose of fentanyl used (mcg/kg)	2.88 ± 0.53	2.95 ± 0.66	0.671

VAS pain score at 30 min	4.0 ± 0.79	3.87 ± 0.73	0.499

	**Number (%)**	**Number (%)**	**p** **-value**

Gender (male)	12 (40%)	10 (33%)	0.593
Gender (female)	18 (60%)	20 (66%)	

ASA PS I	14 (46%)	17 (57%)	0.437
ASA PS II	16 (54%)	13 (43%)	

Smoker	17 (57%)	17 (57%)	1.0

*Note:* Data are presented as numbers (percentage) (*n* (%)), mean ± standard deviation (SD), standard error (SE), or with a 95% confidence interval (CI). A *p* value of < 0.05 was considered statistically significant.

Abbreviations: ASA PS, American Society of Anesthesiology Physical Status; BMI, body mass index; CI, confidence interval; SD, standard deviation; SE, standard error.

Table 2 indicates significant differences in expiratory muscle function, as measured by TFIO during cough, between the two groups. The Sugammadex group exhibited significantly better TFIO values at TOFR 0.9, with a substantial effect size. In contrast, no significant differences were observed between groups at baseline or after 30 min in the PACU.

**TABLE 2:** Expiratory muscle function presented by the thickening fraction of the internal oblique muscle (TOFI) during cough.

	Sugammadex group (*n* = 30)s	Neostigmine group (*n* = 30)	*p* value
	Mean ± SD	Mean ± SD	
TFIO baseline (mm)	9.07 ± 1.93	8.7 ± 2.41	0.518
TFIO to TOFR 0.9 (mm)	8.23 ± 1.78	7 ± 1.93	< 0.05^∗^
TFIO (% change from baseline) at TOFR ≥ 0.9	9.13 ± 6.25	19.32 ± 5.75	< 0.001^∗^
TFIO after 30 min (mm)	8.73 ± 1.86	8.17 ± 2.1	0.273
TFIO (% change from baseline) at 30 min in PACU	3.55 ± 5.25	5.38 ± 5.28	0.182

	**Number (%)**	**Number (%)**	**p** **-value**

No patients returned to baseline TFIO at TOFR 0.9	8.0 (27%)	0.0 (0%)	< 0.001^∗^
No patients returned to baseline TFIO 30 min in PACU	20.0 (67%)	14.0 (47%)	1.118

*Note:* Data are presented as numbers (percentage), mean ± standard deviation (SD), standard error (SE), or 95% confidence interval (CI). Statistical significance was set at a *p* value < 0.05. Effect sizes were interpreted as follows: Cohen’s d^∗^: 0.2 = small, 0.5 = medium, 0.8 = large; Cramér’s V: 0.00–0.10 = negligible, 0.10–0.30 = small, 0.30–0.50 = medium, ≥ 0.50 = large. min, minutes; TFIO, thickening fraction of the internal oblique muscle; ΔTFIO, percentage change in TFIO from baseline to TOFR ≥ 0.9 or 30 min after PACU arrival, calculated as follows.

Abbreviations: CI, confidence interval; PACU, postanesthesia care unit; SD, standard deviation; SE, standard error; TOFR, train‐of‐four ratio.

Table 3 and Figure 6 demonstrate that baseline DE was comparable between groups. Nevertheless, significant differences emerged at subsequent time points. Sugammadex was associated with a notably greater percentage reduction in DE (ΔDE%) at a TOFR of 0.9 on the right side, indicating a beneficial effect on the recovery of inspiratory muscle function.

**TABLE 3:** Comparison of diaphragmatic excursion (DE) measurements between the sugammadex and neostigmine groups at baseline, TOFR ≥ 0.9, and 30 min in the PACU.

		Sugammadex group (*n* = 30)	Neostigmine group (*n* = 30)	*p* value
		Mean ± SD	Mean ± SD	
Right diaphragm	Right DE (Baseline)	6.49 ± 0.64	6.41 ± 0.62	0.655
	Right DE (TOFR 0.9)	4.60 ± 0.34	4.07 ± 0.40	< 0.001^∗^
	Right ΔDE (at TOFR 0.9) (%)	1.91 ± 0.66	2.34 ± 0.52	< 0.05^∗^
	Right DE (after 30 min)	5.06 ± 0.35	4.96 ± 0.34	0.249
	Right ΔDE (after 30 min) (%)	1.42 ± 0.49	1.64 ± 0.98	0.284

		**Number (%)**	**Number (%)**	*p* **value**

	No patients returned to the baseline value of DE at TOFR 0.9	0.0 (0.0%)	0.0 (0.0%)	1.0
	No patients returned to the baseline value of DE after 30 min in PACU	0.0 (0.0%)	0.0 (0.0%)	1.0

*Note:* Data are presented as numbers (percentage) (*n* (%)), mean ± standard deviation (SD), standard error (SE), or 95% confidence interval (CI). A *p* value < 0.05 was considered statistically significant. min, minutes; ΔDIA, absolute change in diaphragm excursion from baseline.

Abbreviations: CI, confidence interval; DE, diaphragmatic excursion; PACU, postanesthesia care unit; SD, standard deviation; SE, standard error; TOFR, train‐of‐four ratio.

^∗^DE were significantly improved with sugammadex at TOFR ≥ 0.9, these differences did not persist after 30 min. This indicates that neuromuscular recovery continues over time, regardless of the reversal agent used.

**FIGURE 6:**
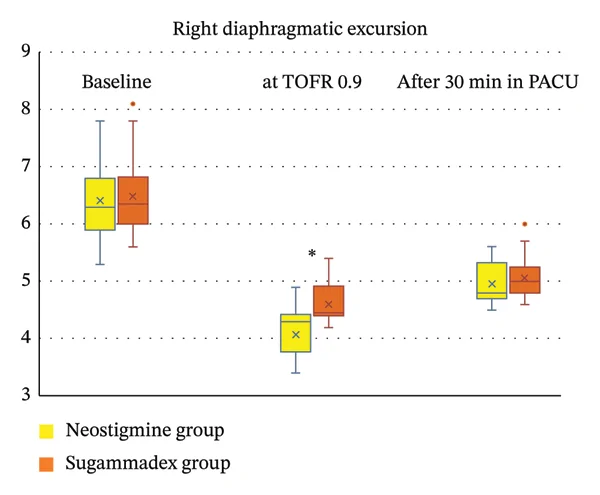
The figure compares sugammadex results with neostigmine results regarding right DE at baseline and TOFR 0.9, as well as after 30 min in the PACU.

Table 4 displays the time intervals from the administration of neostigmine and sugammadex until the achievement of TOFR of 0.9, 0.95, and 1.0. The data indicate that the sugammadex group showed significantly shorter time intervals than the neostigmine group across all endpoints, underscoring its more rapid reversal of neuromuscular blockade. The substantial effect sizes demonstrate that these differences are both statistically significant and clinically meaningful, highlighting the greater efficacy of sugammadex.

**TABLE 4:** Time intervals from reversal agent administration to achievement of train‐of‐four ratios (TOFRs) of 0.9, 0.95, and 1.0.

	Sugammadex group (*n* = 30)	Neostigmine group (*n* = 30)	*p* value
	Mean ± SD	Mean ± SD	
Time from reversal to TOFR 0.9 (min)	4.87 ± 0.86	12.2 ± 1.85	< 0.001^∗^
TOFR. 0.90–95 (min)	0.32 ± 0.11	1.74 ± 0.66	< 0.001^∗^
TOFR. 0.90–1.00 (min)	0.53 ± 0.17	5.13 ± 1.31	< 0.001^∗^

*Note:* Data are presented as mean ± standard deviation (SD), standard error (SE), or 95% confidence interval (CI). Statistical significance was set as a *p* value < 0.05. Cohen’s d^∗^ effect sizes were interpreted as follows: 0.2 = small, 0.5 = medium, and 0.8 = large.

Abbreviations: CI, confidence interval; SD, standard deviation; SE, standard error; TOFR, train‐of‐four ratio.

Despite the absence of significant differences between groups at 30 min, both groups continued to demonstrate reduced TFIO and DE compared to baseline values, indicating incomplete recovery of respiratory muscle function.

No statistically significant differences were detected between the two groups in the incidence of pulmonary complications, including (*p* = 0.447), hypoxia (*p* = 0.228), and reoxygenation (*p* = 0.313). Notably, no cases of reintubation, airway obstruction, pulmonary edema, or clinical signs of postoperative residual paralysis were recorded in either group.

Table 5 displays that the incidence of atelectasis was 10% (3/30) in the sugammadex group compared to 16.7% (5/30) in the neostigmine group, corresponding to an absolute risk reduction (ARR) of 6.7% and a relative risk (RR) of 0.60, indicating a 40% relative risk reduction. The incidence of hypoxia was 6.7% (2/30) in the sugammadex group versus 16.7% (5/30) in the neostigmine group, yielding an ARR of 10% and an RR of 0.40 (60% relative risk reduction). Tachycardia occurred in 0% of patients in the sugammadex group compared to 40% (12/30) in the neostigmine group, corresponding to an ARR of 40% and an RR approaching 0, indicating a substantial reduction in risk with sugammadex.

**TABLE 5:** Absolute and relative effect sizes for binary outcomes.

Outcome	Sugammadex (*n* = 30)	Neostigmine (*n* = 30)	Absolute risk reduction (ARR)	Relative risk (RR)	Relative risk reduction (RRR)	NNT
Atelectasis	3 (10%)	5 (16.7%)	6.7%	0.60	40%	15
Hypoxia	2 (6.7%)	5 (16.7%)	10%	0.40	60%	10
Tachycardia	0 (0%)	12 (40%)	40%	0.00^∗^	100%	3

*Note:* ARR (absolute risk reduction) = control–treatment; RR (relative risk) = treatment/control; RRR (relative risk reduction) = 1–RR; NNT (number needed to treat) = 1/ARR.

No statistically significant difference was observed between groups in the incidence of adverse drug effects (*p*‐value = 0.641) or postoperative nausea and vomiting (PONV; *p*‐value 0.447). In contrast, tachycardia occurred significantly more frequently in the neostigmine group (*p*‐value < 0.001).

Tables 6 and 7 depict a comparison between the two study groups regarding complications and drug side effects. The analysis indicates that although there are differences between the groups concerning specific complications, only tachycardia demonstrated a statistically significant association with the neostigmine group.

**TABLE 6:** Comparison between groups in terms of complications.

Pulmonary complications	Sugammadex group (*n* = 30)	Neostigmine group (*n* = 30)	*p* value
	Number (%)	Number (%)	
Incidence of atelectasis	3.0 (10)	5.0 (16.7)	0.447
Low SpO_2_ in PACU	2.0 (6.7)	5.0 (16.7)	0.228
Reintubation	0.0 (0.0)	0.0 (0.0)	1.0 £
Recurarization	0.0 (0.0)	1.0 (3.3)	0.313
Airway obstruction	0.0 (0.0)	0.0 (0.0)	1.0 £
Negative pressure pulmonary edema	0.0 (0.0)	0.0 (0.0)	1.0 £
Sign of postoperative residual paralysis	0.0 (0.0)	0.0 (0.0)	1.0 £

*Note:* Data are presented as numbers (%). Statistical significance is considered if the *p* value < 0.05. Fisher’s data are presented as numbers (percentages). Statistical significance was defined as a *p* value < 0.05. £ Fisher’s exact test was used for comparisons where expected cell frequencies were less than 5, as the chi‐squared test is not valid under these conditions. The strength of association for significant findings was interpreted using Cramér’s V: 0.00–0.10 = negligible effect, 0.10–0.30 = small effect, 0.30–0.50 = medium effect, and ≥ 0.50 = large effect. SpO_2_, peripheral oxygen saturation.

Abbreviations: PACU, postanesthesia care unit; PONV, postoperative nausea and vomiting.

**TABLE 7:** Comparison between groups regarding drug side effects.

Drug side effects	Sugammadex group (*n* = 30)	Neostigmine group (*n* = 30)	*p* value
	Number (%)	Number (%)	
PONV	3.0 (10%)	5.0 (16.7%)	0.447
Adverse drug effect	2.0 (6.6%)	3.0 (10%)	0.641
Tachycardia	0.0 (0.0%)	12.0 (40%)	< 0.001^∗^

*Note:* Data are presented as numbers (%). Statistical significance was set at a *p* value < 0.05. Fisher’s exact test was employed for analyses where expected cell frequencies were low, as the chi‐squared test is not valid under such conditions. The strength of statistically significant associations was interpreted using Cramér’s V, with values ranging from 0.00 to 0.10 indicating a negligible effect, 0.10 to 0.30 indicating a small effect, 0.30 to 0.50 indicating a medium effect, and ≥ 0.50 indicating a large effect.

## 6. Discussion

PORC is a significant and frequently underestimated complication. The reported incidence is variable, with some studies reporting rates as high as 57.8% at extubation and 45.2% in the PACU, depending on monitoring techniques and clinical practice [8].

Sugammadex leads to a significantly more rapid recovery of neuromuscular function in comparison to neostigmine. This is demonstrated by the shorter times that were needed to reach a TOFR of 0.9, 0.95, and 1.0. These results were aligned with previous studies and systematic reviews that highlight the quicker and more reliable reversal effects of sugammadex. Additionally, a Cochrane review has indicated that sugammadex results in a lower residual NMB and fewer early postoperative complications compared to neostigmine [3].

Prolonged NMB is associated with critical postoperative respiratory events. It may also be influenced by factors such as extended narcotic effects, patient age, emergency procedures, prolonged surgical duration, and specific types of surgeries, including cavity and vascular surgeries [13].

Accurate assessment of NMB requires quantitative measurement of the TOFR using neuromuscular monitoring devices, complemented by vigilant detection of side effects associated with neuromuscular blocking agents [14].

The primary outcome of the study reveals that the recovery of respiratory muscle function, as assessed through ultrasonographic parameters (TFIO and DE), was significantly better in patients who received sugammadex in comparison to those who received neostigmine. The smaller reductions in TFIO and DE indicate a better preservation of both expiratory and inspiratory muscle functions. These findings suggest that sugammadex is more effective in restoring clinically significant respiratory muscle performance.

Significant residual respiratory muscle dysfunction was still evident, even after achieving a TOFR of 0.9 or even 1.0. In most patients at these thresholds, neither TFIO nor DE returned to baseline values. This highlights a major limitation of TOF monitoring: while it reflects recovery in the adductor pollicis muscle, it may not accurately represent the functional status of respiratory muscles. These findings suggest that relying solely on TOF monitoring could lead to an overestimation of the degree of functional recovery.

These findings emphasize the important role of ultrasonography in assessing neuromuscular recovery. Using ultrasound to evaluate respiratory muscles provides a direct and functional way to monitor neuromuscular function, potentially improving upon traditional monitoring methods. While it cannot completely replace standard monitoring techniques, ultrasonography may offer greater sensitivity in detecting residual neuromuscular impairment. Additional research is needed to determine whether ultrasound‐based assessments can improve the prediction of PPCs and assist in clinical decision‐making.

A systematic review found that sufficient recovery of muscle power and the absence of residual blockade are consistently linked to a TOFR greater than 0.9 [15].

Clinical evaluation, like the sustained head lift, hand grip, and tongue protrusion, is commonly used to assess residual NMB. However, these tests have poor sensitivity and specificity, making objective neuromuscular monitoring essential. Such monitoring is often underutilized due to a lack of awareness and limited availability of appropriate devices [16].

Our study evaluated the effectiveness of sugammadex and neostigmine in reversing NMB by using ultrasound‐based assessments of DE and expiratory muscle strength. This method is suggested to be superior to monitoring TOFR. Sugammadex demonstrated significantly faster recovery, achieving TOFR thresholds (0.9 ⟶ 0.95 and 0.9 ⟶ 1) approximately 7–10 times faster than neostigmine (0.32 vs 1.74 min; 0.53 vs 5.13 min).

Even after 30 min in PACU, respiratory muscle function had not fully returned to baseline levels in either group, even though the TOFR suggested some recovery. This finding emphasizes the ongoing functional impairment that traditional neuromuscular monitoring may fail to identify.

These findings align with previous evidence showing improvements in respiratory parameters (e.g., EMGdi activity, tidal volume, PaO_2_) with sugammadex. This enhanced efficacy is due to its direct encapsulation of rocuronium, unlike neostigmine, which indirectly inhibits acetylcholinesterase [17].

Additional randomized trials support these findings, consistently showing that sugammadex enables faster recovery than neostigmine, even when given during deeper levels of neuromuscular blockade compared to moderate levels [18]. Similarly, Moon et al. (2020) reported significantly shorter recovery times (TOFR ≥ 0.9: 10 vs 40 min; *p* < 0.001) [19]. Similar results were observed in laparoscopic procedures, where sugammadex allowed for quicker recovery from profound blockade without delaying emergence [18, 20].

Regarding diaphragmatic function, sugammadex led to significant improvements in DE at a TOFR of 0.9 or higher, while neostigmine did not show the same effect. However, baseline DE was not completely recovered in any of the patients, even after 30 min in the PACU. This is likely due to impaired diaphragmatic movement caused by pneumoperitoneum during laparoscopic surgery. Additionally, greater reductions in DE were associated with more severe dysfunction of the inspiratory muscles.

These results differ somewhat from those of Huang et al., who detected that 30%–37% of patients regained their baseline diaphragmatic function after receiving sugammadex, whereas none or fewer achieved this with neostigmine. This discrepancy may be due to differences in the types of surgeries performed—microlaryngeal versus laparoscopic—since microlaryngeal surgery is associated with less diaphragmatic impairment [12].

In this study, the expiratory muscle strength recovery, as assessed by TFIO, was limited when the TOFR reached 0.9 or higher. Only 27% of patients in the sugammadex group achieved baseline values, while none in the neostigmine group did. After 30 min in the PACU, recovery improved but remained statistically nonsignificant between the two groups, with rates of 67% for sugammadex and 47% for neostigmine. These results align with the results of Huang et al. (2023), which declared that only 7% of patients in the sugammadex group and 0% in the neostigmine group reached baseline TFIO at a TOFR of 0.9 or greater. After 30 min, these numbers increased to 33% for sugammadex and 14% for neostigmine [12].

While TFIO and DE were significantly improved with sugammadex at TOFR ≥ 0.9, these differences did not persist after 30 min. This indicates that neuromuscular recovery continues over time, regardless of the reversal agent used. This finding is consistent with Huang [12] and Abola et al., where incentive spirometry volumes were comparable between groups at later postoperative intervals [7].

Ultrasound findings were supported by Cappellini et al., who demonstrated enhanced DE at extubation with sugammadex [8]. Schreiber et al. demonstrated that TFIO correlates with cough strength, specifically peak airway pressure, and that reduced TFIO is accompanied by a higher risk of failed extubation [10].

Regarding PPCs, this study did not detect statistically significant differences between the two groups. The risk of atelectasis was 16.7% with neostigmine in comparison to 10% with sugammadex (*p* < 0.447), which contrasts with the results of Yu et al., who declared a significantly lower incidence with sugammadex [21]. Similarly, hypoxia occurred in 16.7% of patients receiving neostigmine versus 6.7% with sugammadex (*p* < 0.228), differing from Moon et al., where no hypoxic episodes were observed in the sugammadex group [19]. Recurarization rates were low and not significantly different (3.3% vs. 0%; *p* < 0.313), consistent with the findings of Ghoneim and El Beltagy [22].

Overall adverse effects were also comparable between groups (10% for neostigmine vs. 6.6% for sugammadex). However, subgroup analysis declared a significant increase in the incidence of tachycardia in the neostigmine group (40% vs. 0%), likely attributable to the concomitant use of atropine, in agreement with previous findings [20]. In contrast, the incidence of PONV did not show a significant difference between groups (16.7% vs. 10%), aligned with Abad‐Gurumeta et al. results [23].

#Our results both complement and diverge from those reported by Huang et al., who first applied TFIO and DE to compare sugammadex and neostigmine in a microlaryngeal surgery population. In their trial, a subset of patients receiving sugammadex regained baseline DE and TFIO within 30 min after extubation, whereas recovery was slower and less complete in the neostigmine group. In contrast, none of the patients in this study achieved full restoration of DE at TOFR ≥ 0.9 or at 30 min in the PACU, and the proportion reaching baseline TFIO remained limited in both groups, despite a clear advantage for sugammadex [9].

These discrepancies are likely attributable to differences in surgical context and respiratory mechanics. Huang et al. studied patients undergoing microlaryngeal procedures, where diaphragmatic mechanics are relatively preserved. Our cohort underwent laparoscopic cholecystectomy with pneumoperitoneum, which is known to reduce diaphragmatic mobility and alter abdominal wall dynamics, potentially prolonging respiratory muscle dysfunction even after neuromuscular transmission at the adductor pollicis appears normal. Thus, although both trials show superior early recovery of TFIO and DE with sugammadex, our findings suggest that residual respiratory muscle impairment may persist longer in abdominal laparoscopic surgery [9].

Furthermore, the magnitude of TFIO reduction from baseline to TOFR ≥ 0.9 was greater in our neostigmine group than in Huang’s study, and we observed a markedly higher incidence of tachycardia associated with neostigmine‐atropine. These differences underscore that the clinical impact of reversal strategy may be modulated by the type of surgery, the extent of pneumoperitoneum, and the underlying cardiopulmonary risk profile of the population studied [9].

### 6.1. Study Limitations

The current study has several limitations. The tests employed were voluntary assessments (TFIO during cough and DE during maximum sniff breathing), necessitating patient cooperation. Postoperative pain can negatively affect performance, especially in abdominal surgeries, as shown in this study, potentially leading to decreased measurements of postoperative muscle strength. However, this limitation can be mitigated by opting for laparoscopic surgery rather than open surgery.

One significant limitation of this study is the relatively small sample size, which may have led to insufficient statistical power to identify differences in various clinical outcomes. This is especially relevant for PPCs and recovery endpoints measured at 30 min in the PACU. Although many of these outcomes did not reach statistical significance, consistent trends favoring sugammadex were observed, including lower incidences of atelectasis, hypoxia, recurarization, postoperative nausea and vomiting, and improved recovery of respiratory muscle function. Therefore, the absence of statistically significant differences should be interpreted cautiously, as it may reflect underpowering rather than true equivalence between the two groups. Larger multicenter studies are warranted to confirm these findings and better evaluate clinically relevant postoperative outcomes.

## 7. Conclusion

Sugammadex results in more effective expiratory muscle function recovery compared to neostigmine at TOFR ≥ 0.9. However, significant residual respiratory muscle dysfunction persists despite achieving TOFR ≥ 0.9, indicating that TOF monitoring alone may be insufficient to detect incomplete respiratory muscle strength recovery. Ultrasonographic assessment of respiratory muscles appears to provide additional functional information and may be a more sensitive detector for residual neuromuscular impairment.

## Funding

This research did not receive external funding and was carried out as part of an academic thesis at the Faculty of Medicine, Ain Shams University.

## Conflicts of Interest

The authors declare no conflicts of interest.

## Data Availability

The data that support the findings of this study are available on request from the corresponding author. The data are not publicly available due to privacy or ethical restrictions.
